# Five critically ill pregnant women/parturients treated with extracorporeal membrane oxygenation

**DOI:** 10.1186/s13019-022-02093-1

**Published:** 2022-12-18

**Authors:** Ying Li, Chi Xu, Furong Li, Zheng Yan, Shugao Ye, Jinqi Ma, Juan Wen

**Affiliations:** 1 Department of Obstetrics and Gynecology, The Affiliated Wuxi People’s Hospital of Nanjing Medical University, Wuxi, China; 2 Emergency Department, The Affiliated Wuxi People’s Hospital of Nanjing Medical University, Wuxi, China; 3 Department of Critical Care Medicine, The Affiliated Wuxi People’s Hospital of Nanjing Medical University, Wuxi, China; 4 Department of Thoracic Surgery, The Affiliated Wuxi People’s Hospital of Nanjing Medical University, Wuxi, China; 5 Nanjing Maternity and Child Health Care Institute, Women’s Hospital of Nanjing Medical University, Nanjing Maternity and Child Health Care Hospital, Nanjing, China

**Keywords:** Critically ill pregnant women/parturients, Extracorporeal membrane oxygenation, Postpartum hemorrhage, Respiratory and circulatory failure, China

## Abstract

**Background:**

Maternal mortality has always been a major medical concern. Recently, the successful application of extracorporeal membrane oxygenation (ECMO) technology in the rescue of near-death patients has been reported.

**Case presentation:**

This study retrospectively analyzed 5 cases of critically ill pregnant women/parturients treated with ECMO for respiratory and circulatory failure in the Wuxi People’s Hospital from 2018 to 2020. The mean age of the 5 cases was 30.2 years. Among them, Cases 1 and 5 were treated with Venoarterial (VA) ECMO. Case 1 was diagnosed with congenital heart disease, atrial septal defect, and severe pulmonary hypertension. VA ECMO was applied before cesarean section and was successfully removed after double lung transplantation, but the patient died 10 months after delivery from lung infection. While Case 5 was diagnosed with systemic lupus erythematosus, lupus nephritis, thrombotic vascular disease, HELLP syndrome, and cerebral hemorrhage. VA ECMO was applied 39 days after cesarean section, and the patient died 40 days after delivery due to multiple organ failure. Cases 3 and 4 were treated with Venovenous (VV) ECMO. Case 3 was diagnosed with refractory postpartum hemorrhage, and Case 4 was diagnosed with postpartum hypoglycemic coma, aspiration pneumonia, and shock. They were treated with VV ECMO after delivery, and all survived after successful evacuation. Another Case (Case 2) was diagnosed with postpartum pelvic infection, sepsis and septic shock, and was treated with VA ECMO at 15 days after delivery. The patient changed to VV ECMO at 30 days after delivery due to significant improvement in heart function and poor lung function, but eventually died of multiple organ failure. For the 5 cases, the mean duration of ECMO was 8.7 days, the mean duration of intensive care was 22.0 days, and the mean length of hospital stay was 57.6 days. As a result, 3 patients gradually returned to normal with significant improvement in ventilation and oxygenation after ECMO treatment.

**Conclusions:**

ECMO technology can be used to treat some of the critical obstetric patients with respiratory and circulatory failure that is ineffective to conventional treatment, but it has no therapeutic effect on the primary disease.

## Background

Maternal mortality has always been a major medical concern. In recent 20 years, the successful application of Extracorporeal Membrane Oxygenation (ECMO) technology in the rescue of near-death patients has been reported year by year [1–5]. According to statistics of Extracorporeal Life Support Organization (ELSO), as of October 19, 2021, there were 154,106 patients treated with ECMO worldwide [6]. However, ELSO does not report specific statistical data related to the application of ECMO in pregnant women or parturients. A recent systematic review has quantified the number of cases and indications for extracorporeal life support (ECLS) in women during the peripartum period from 221 studies and reported maternal and fetal complications and outcomes associated with peripartum ECLS [7], but few from Chinese women. In the past decade, there have been reports of successful treatment of acute respiratory distress syndrome (ARDS) in pregnant women complicated with influenza A (H1N1) in China after application of ECMO [8, 9]. This study retrospectively analyzed the data of 5 critically ill pregnant women/parturients treated by ECMO technology, summarized the treatment process and complications, and provided reference for the application of ECMO technology in critically ill pregnant women/parturients.

### Case presentation

This study retrospectively included 5 cases of critically ill pregnant women/parturients treated with ECMO for respiratory and circulatory failure in the Affiliated Wuxi People’s Hospital of Nanjing Medical University from January 2018 to December 2020, all of whom had indications of ECMO treatment without contraindications [10]. Wuxi People's Hospital is one of the leading lung transplant centres in the world, with mature ECMO technology. The 5 critically ill pregnant women/parturients on the verge of death underwent active rescue and decided to apply the ECMO technique, which was jointly completed by the ECMO team of thoracic surgery, doctors from the department of critical care medicine and anesthesiologists. Venoarterial (VA) ECMO or Venovenous (VV) ECMO are selected according to therapeutic purposes, and standard procedures are followed for catheterization, monitoring, and withdrawal of the ECMO [11, 12]. The study was approved by the institutional review board of Wuxi People's Hospital, and written informed consent were obtained from the patient or patient's relative.

### Basic information of 5 cases before ECMO application (***Table ******1***)

**Table 1:** Clinical characteristics and ECMO indications of 5 cases

Case	Age (year)	Gravidity/parity	Delivery mode	Application time of ECMO	Primary illness	ECMO indications	Number of CPR performed prior to ECMO
Case 1	42	7/1	Caesarean section	27 Weeks and 5 days of gestation	Severe pulmonary hypertension, congenital heart disease, atrial septal defect	Right heart failure	0
Case 2	28	1/1	Vaginal delivery	Postpartum 15 days	Pelvic infection, puerperal sepsis	Septic shock, cardiogenic shock	1
Case 3	27	5/2	Vaginal delivery	The day of delivery	Refractory postpartum hemorrhage	ARDS	2
Case 4	31	4/1	Vaginal delivery	Postpartum 24 days	Type I diabetes, postpartum hypoglycemia	ARDS	2
Case 5	23	3/1	Caesarean section	Postpartum 39 days	Systemic lupus erythematosus, lupus nephritis	Severe myocardial depression, circulatory failure	2

ECMO, extracorporeal membrane oxygenation; CPR, cardiopulmonary resuscitation; ARDS, acute respiratory distress syndrome

Case 1 was diagnosed with congenital heart disease, secundum atrial septal defect (central type), and severe pulmonary hypertension (mean pulmonary artery pressure: 104 mmHg) in 2013. During the early pregnancy, a multi-disciplinary team in our hospital recommended termination of pregnancy, but the patient refused. The patient was admitted to hospital at 21 weeks and 3 days of gestation (May 3, 2018) with NYHA grade III cardiac function, and mean pulmonary artery pressure of 140 mmHg as indicated by echocardiography. At 27 weeks and 5 days of gestation, her condition worsened, and she was given continuous high flow oxygen (inhaled oxygen flow 35 L/min, oxygen concentration 95%). Blood oxygen saturation was maintained at 0.82–0.88 and heart rate was 116 beats /min. On the same day, emergency application of ECMO followed by cesarean section.

Case 2 delivered vaginally at 38 weeks and 6 days (June 2018) in another hospital. The delivery process was smooth, and left perineal incision and suture were performed during delivery. At 7 days postpartum, the woman developed lower abdominal pain and fever. At 14 days postpartum, B-ultrasonography showed cystic mass in the right ovary with pyometra in the right salpinx. After anti-inflammatory therapy, there was no improvement and blood pressure tended to decrease. At 15 days postpartum, laparoscopic right salpingectomy, pelvic abscess removal and pelvic adhesiolysis were performed in emergency, and the pus during the operation indicated gram-positive cocci. Postoperative trachea extubation was performed with clear consciousness. Ten minutes after extubation, there was confusion and decreased blood pressure. The patient was immediately rescued with pressure boost, capacity enlargement, blood transfusion and endotracheal intubation, and transferred to our hospital. ECMO was urgently applied in our hospital for sepsis, septic shock, and multifunctional organ dysfunction syndrome (circulatory and respiratory).

Case 3 delivered vaginally at 40 weeks and 5 days of gestation (December 2018) in township hospital. Due to refractory postpartum hemorrhage, disseminated intravascular coagulation and cardiac arrest, cardiopulmonary resuscitation and total hysterectomy were performed immediately. After surgery, she was transferred to our hospital. Due to intra-abdominal bleeding, the right uterine vein ligation and bilateral internal iliac artery ligation were performed, and the postpartum hemorrhage totaled 5000 ml. At the end of the second operation, the heartbeat dropped to 30 beats /min again. After cardiopulmonary resuscitation, ECMO was urgently applied due to poor oxygenation.

Case 4 was diagnosed with type I diabetes mellitus in 1999 and received long-term subcutaneous insulin treatment. On July 24, 2019, she delivered vaginally at 38 weeks and 6 days of gestation in our hospital. After delivery, the dosage of insulin was adjusted to 1/3 of that before delivery, and blood glucose was controlled within the target range during hospitalization. At 24 days after delivery, the blood glucose was measured at home as "Low", and dizziness, unconsciousness and coma appeared. The woman was still unconscious after her family fed her with about 250 ml sugar water, and then she was hospitalized in emergency. The monitored blood oxygen saturation value was 0.09, and endotracheal intubation and ventilator assisted ventilation were immediately given. Oxygen saturation did not rise after rising to 0.50, and there were two episodes of slow heart rate (< 40 beats/min). Then, ECMO was applied immediately.

Case 5 was diagnosed with systemic lupus erythematosus and polymyositis in 2017, and was successively treated with hormone, tacrolimus and methotrexate. After January 2020, the patient stopped using tacrolimus and methotrexate without following the doctor's advice and became pregnant (last menstruation: March 21, 2020). During pregnancy, the patient received hydroxychloroquine, aspirin, enoxaparin, and prednisone (5 mg per day (mg/day)). At 24 weeks of gestation, prednisone was added to 30 mg/day due to increased urinary protein, positive antinuclear antibody and anti-U1 ribonucleoprotein. At 25 weeks and 4 days of gestation, the SLEDA score was 7, and the patient was given methylprednisolone 120 mg/day, along with immunotherapy with gamma globulin and cyclosporine. At 25 weeks and 5 days of gestation, she was suddenly unconscious, with 8 breaths/min and oxygen saturation of 88%. CT examination suggested multiple cerebral hemorrhage and diffuse cerebral edema. On the same day, due to cerebral hemorrhage, cerebral hernia and HELLP syndrome, emergency caesarean delivery was performed. After operation, ventilator-assisted ventilation, anti-infection, diuresis, hormone shock, plasmapheresis and other treatments were given. At 39 days postpartum, the blood pressure decreased progressively, and ECMO was urgently applied.

### ECMO treatment and outcome of the 5 cases (***Table ******2***)

**Table 2:** ECMO treatment and outcome of the 5 cases

Case	Conventional rescue time (h)	ECMO type	Duration of ECMO (d)	Duration of intensive care (d)	Hospital stays (d)	Other treatment	Transfusion of blood and blood products	Complications	Maternal and neonatal outcomes
Case 1	4	VA	16	25	139	VAV, atrial septal defect repair, double lung transplantation, tracheotomy	SLRRBC (14 U), plasma (2500 ml), platelet (1 dose)	Thrombus at the MO, local stratification and plasma penetration of the MO, pulmonary infection, inferior vena cava thrombosis	Mother died at 10 months after delivery and newborn survived
Case 2	7	VA → VV	22	22	22	Blood purification, VAV, anti-infection, acid suppression, nutritional support	SLRRBC (41 U), plasma (7550 ml), CCF (40 U), platelet (84 dose)	Ventilator-associated pneumonia, coagulopathy, multiple organ dysfunction syndrome, central diabetes insipidus, brain death, post-cardiopulmonary resuscitation syndrome, liver insufficiency, microbubbles in ECMO tubes	Mother died and newborn survived
Case 3	6	VV	2	18	65	VAV, continuous veno-venous hemofiltration (CVVH)	SLRRBC (40 U), plasma (4900 ml), CCF (10 U), platelet (2 dose)	Pulmonary infection, Sheehan's syndrome, hypopituitary crisis, acute hepatic and renal insufficiency, right lower limb ischemia, postpartum depression	Both mother and newborn survived
Case 4	4	VV	3	6	18	VAV	SLRRBC (3 U), plasma (400 ml)	Pulmonary infection	Both mother and newborn survived
Case 5	17	VA	1/2	39	44	VAV, hormone shock, immunoregulation, dehydration, tracheotomy, continuous renal replacement therapy (CRRT)	SLRRBC (18 U), plasma (5525 ml), platelet (9 dose)	Pulmonary infection, pulmonary edema, acute renal insufficiency, hypothyroidism, severe metabolic acidosis, pancreatitis, coagulopathy	Both mother and newborn died

ECMO, extracorporeal membrane oxygenation; VAV, ventilator-assisted ventilation; SLRRBC, suspended leukocyte-reduced red blood cells; CCF, cryoprecipitate coagulation factor; MO, membrane oxygenation

For case 1, VA ECMO was first used, and then cesarean section, ligation of bilateral uterine artery ascending branches, ligation of bilateral ovarian artery communicating branches, and ligation of bilateral fallopian tubes were performed. After surgery, the patient was treated with ventilator assisted ventilation, ECMO cardiopulmonary support, pulmonary hypertension control, anti-infection and diuretic treatment, and the patient had good uterine contraction and less lochia. Since then, many attempts to remove ECMO have failed, with poor cardiac function. At 11 days after the cesarean section, repair of atrial septal defect and double lung transplantation were performed. The operation was successful, and ECMO was successfully removed at 5 days after the operation (16 days after the cesarean section). However, the patient developed a pulmonary infection 10 months after the operation due to self-discontinuation of anti-infective and immunosuppressive drugs, and died on April 1, 2019 (287 days after the cesarean section) despite active treatment. At present, the newborn is developing well without neurological abnormalities. Case 2 was treated with VA ECMO at 15 days after delivery. The patient changed to VV ECMO at 30 days after delivery due to significant improvement in heart function and poor lung function, but eventually died of multiple organ failure due to pelvic infection and sepsis. Cases 3 and 4 were treated with VV ECMO after delivery, and were successfully evacuated from ECMO and survived. Case 5 died of multiple organ failure and circulation deterioration even after VA ECMO was applied at 39 days after delivery. For the 5 cases, the mean duration of ECMO was 8.7 days, the mean duration of intensive care was 22.0 days, and the mean length of hospital stay was 57.6 days.

### Coagulation conditions during ECMO treatment

Heparin was used for anticoagulation during ECMO treatment, and the pump speed of heparin was adjusted according to the target values (activated clotting time (ACT): 130–150 s; activated partial thromboplastin time (APTT): 50–60 s). For the 3 patients (Cases 1, 3 and 4) who successfully treated by ECMO, the ACT and APTT were maintained in the range of 73.7–142.0 s and 26.4–51.0 s, respectively. While for the 2 patients (Cases 2 and 5) who treated by ECMO unsuccessfully, the ACT and APTT were maintained in the range of 126.0–154.0 s and 32.5–86.8 s, respectively (Table 3). Among the 5 patients, only Case 1 developed thrombotic complications (thrombus at the membrane oxygenation and inferior vena cava thrombosis), which may be related to ECMO type, hemodynamic changes caused by the primary disease, duration of ECMO use, infection and other factors.

**Table 3:** Coagulation conditions of the 5 cases during ECMO treatment

Case	Anticoagulant	ACT (s)	APTT (s)
Case 1	Heparin	73.7–109.0	34.1–51.0
Case 2	Heparin	140.0–154.0	32.5–76.5
Case 3	Heparin	96.0–115.2	35.0–43.6
Case 4	Heparin	98.3–142.0	26.4–45.5
Case 5	Heparin	126.0–143.0	64.3–86.8

ECMO, extracorporeal membrane oxygenation; ACT, activated clotting time; APTT, activated partial thromboplastin time

## Discussion and conclusions

ECMO, also known as artificial heart and lung, is an effective in vitro life support technology for patients with extremely critical cardiopulmonary failure. Its working principle is to realize the exchange of gas and blood through the extracorporeal circulation system, partially replace pulmonary oxygenation and cardiac pump function, significantly improve the patient's oxygenation and ventilation, correct hypoxemia and hypercapnia, and ultimately achieve the recovery of cardiopulmonary function [11]. When the primary disease becomes acute or progressive and cannot be sustained by conventional means, ECMO can be used. It can provide temporary cardiopulmonary support for shock patients and is increasingly used in intensive care medicine. ECMO can be used continuously for hours to days and it has been reported that ECMO can be used for peripartum patient up to 110 days [13].

In the application of ECMO in pregnant women/parturients, there are both obstetric and non-obstetric factors. In developed countries, ECMO technology has been increasingly reported in the treatment of pregnant women/parturients with acute respiratory failure, severe heart failure, postpartum hemorrhage induced cardiac arrest, pulmonary embolism, and amniotic fluid embolism [14–16]. Ong et al. systematically reviewed ECMO indications of 97 pregnant women/parturients and found that adult respiratory distress syndrome (91.9%) was the most common respiratory tract indication, while pulmonary embolism (23.7%) and perinatal cardiomyopathy (16.9%) were the two most common cardiovascular indications [17]. In this study, indications of ECMO included refractory postpartum hemorrhage, puerperal infection, congenital heart disease with severe pulmonary hypertension, HELLP due to systemic lupus erythematosus, and severe hypoglycemic coma. In the 5 cases treated with ECMO, 3 patients gradually returned to normal with significant improvement in ventilation and oxygenation, while the other 2 patients failed to respond to treatment and eventually died.

Due to the risk of thrombosis and embolism during ECMO application, heparin anticoagulation is required regardless of VV ECMO or VA ECMO, so bleeding is the primary complication. Once the bleeding caused by abnormal coagulation function occurs during pregnancy, it will increase the difficulty of treatment, and even endanger the life of pregnant women and fetuses in serious cases. If heparin is used during delivery, it is easy to bleed and coagulation indicators are difficult to monitor due to the surgical incision or placental uterus dissection has not been healed. However, in the 3 patients successfully treated in this study, lochia and other bleeding did not increase due to heparin, and the reasons were considered as follows: (1) Active and effective uterine devascularization was performed during cesarean section (Case 1), and bilateral internal iliac artery ligation was performed during the second operation (case 2); (2) Timely supplement of blood products; (3) Postoperative continuous monitoring of coagulation function, and strictly controlling the dosage of heparin; (4) The use of drugs to promote uterine contractions; (5) It may be related to the hypercoagulability of maternal blood. Therefore, the use of several preventive measures can reduce the risk of severe bleeding after maternal ECMO use. In the other 2 patients who failed to respond to ECMO treatment, coagulation time was more prolonged, which was mainly related to systemic coagulation dysfunction caused by the primary disease.

Our report has several limitations. The current data come from case series with considerable heterogeneity. We did not have an unexposed group without ECMO to compare outcomes. Another limitation is the lack of detailed information on fetal outcomes. Based on these data alone, accurate prediction of fetal risk by maternal ECMO is challenging. Prospective and detailed reporting with multicenter studies may better elucidate the association of ECMO use during pregnancy with maternal and fetal outcomes.

Despite these limitations, we concluded that, ECMO technology can be used to treat some of the critical obstetric patients with respiratory and circulatory failure that is ineffective to conventional treatment. When pregnant women/parturients appear respiratory and circulatory failure, based on active treatment of the primary disease, if there are indications of ECMO, ECMO can be applied as soon as possible [18, 19], and primary care institutions should immediately refer patients to medical institutions with conditions for ECMO application.

At present, ECMO technology, as the ultimate supportive treatment of critical diseases, has complex operation and high requirements. There is still a lack of relevant guidelines or expert consensus on ECMO in the treatment of critically ill pregnant women, and few medical institutions are qualified to use ECMO, so the application and management of ECMO need further research and discussion.

## Data Availability

All datasets generated for this study are included in the manuscript.

## Abbreviations

ECMO: Extracorporeal membrane oxygenation
ELSO: Extracorporeal Life Support Organization
ECLS: Extracorporeal life support
ARDS: Acute respiratory distress syndrome
H1N1: Influenza A
VA: Venoarterial
VV: Venovenous
mg/d: Milligram per day
ACT: Activated clotting time
APTT: Activated partial thromboplastin time
